# Reproductive outcomes following open maternal—fetal surgery for myelomeningocele closure: analysis of MOMS trial participants

**DOI:** 10.1016/j.ajogmf.2025.101765

**Published:** 2025-08-29

**Authors:** Julie S. Moldenhauer, Cora MacPherson, Elizabeth A. Thom, Amy J. Houtrow, N. Scott Adzick

**Affiliations:** Richard D. Wood Jr. Center for Fetal Diagnosis and Treatment, Children’s Hospital of Philadelphia, Philadelphia, PA (Moldenhauer and Adzick); Biostatistics Center, Milken Institute School of Public Health, George Washington University, Washington, DC (MacPherson and Thom); Departments of Physical Medicine and Rehabilitation and Pediatrics, University of Pittsburgh, Pittsburgh, PA (Houtrow).

**Keywords:** fetal surgery, gynecology, infertility, MOMS trial, myelomeningocele, open maternal–fetal surgery, spina bifida, subsequent pregnancy, cirugía fetal, ginecología, infertilidad, ensayo MOMS, mielomeningocele, cirugía materno-fetal abierta, espina bífida, embarazo subsecuente

## Abstract

**BACKGROUND:**

In utero closure of myelomeningocele has become an accepted alternative in the management of prenatally diagnosed spina bifida. Maternal reproductive risk has been previously described on the basis of registry data and institutional outcomes. Herein we aim to provide maternal reproductive outcomes from participants in the Management of Myelomeningocele Study.

**OBJECTIVE:**

Open maternal–fetal surgery for in utero closure of myelomeningocele is associated with childhood benefits through school age; however, obstetrical and maternal reproductive risks are also factors to consider. The objective of this analysis was to evaluate reproductive outcomes after open maternal–fetal surgery compared with standard postnatal myelomeningocele closure.

**STUDY DESIGN:**

The Management of Myelomeningocele Study was a randomized trial comparing prenatal vs postnatal closure of myelomeningocele. Women in the prenatal closure arm underwent open maternal–fetal surgery at 21 to 26 weeks of gestation and underwent cesarean delivery at 37 weeks if they were not delivered before that. In the postnatal closure arm, cesarean delivery was performed at 37 weeks and neonates underwent surgical closure soon after birth. Families returned for follow-up at 30 months and again at school age between 6 and 10 years. Maternal reproductive questionnaires were administered at the time of the follow-up visits to assess menstruation, fertility, gynecologic issues, and subsequent pregnancy outcomes. We compared continuous variables using the Wilcoxon test and categorical variables using the chi-square or Fisher exact test, as appropriate.

**RESULTS:**

A total of 174 randomized women (86 in the prenatal closure group and 88 in the postnatal closure group) completed reproductive questionnaires, with 91 women reporting no subsequent pregnancies since the Management of Myelomeningocele Study. In the prenatal closure group, 36 women reported 60 subsequent pregnancies, of which 45 (75%) progressed ≥20 weeks. In the postnatal closure group, 43 women reported 71 pregnancies, of which 50 (70%) progressed ≥20 weeks. The prenatal closure group was more likely to deliver at <37 weeks (*P*<.001). One uterine rupture (2.2%) and 2 uterine dehiscence cases (4.4%) were reported in the prenatal closure group; none were reported in the postnatal closure group (*P*<.001). The use of fertility treatments or gynecologic surgeries was not different between the groups.

**CONCLUSION:**

Preterm delivery was more common in subsequent pregnancies after open maternal–fetal surgery. The risk for uterine rupture and dehiscence was higher in the prenatal group than in the postnatal group, but lower than what has been previously reported with open maternal–fetal surgery. Reproductive outcomes were otherwise similar between women undergoing open maternal–fetal surgery for myelomeningocele closure and the postnatal closure group in the Management of Myelomeningocele Study. El resumen está disponible en Español al final del artículo.

## Resultados reproductivos después de cirugía materno-fetal abierta para el cierre de mielomeningocele: análisis de participantes del ensayo MOMS

**ANTECEDENTES:**

El cierre intrauterino del mielomeningocele se ha convertido en una alternativa aceptada en el tratamiento de la espina bífida diagnosticada prenatalmente. El riesgo reproductivo materno se ha descrito previamente con base en datos de registros y resultados institucionales. En este estudio, nuestro objetivo es proporcionar resultados reproductivos maternos de los participantes en el Estudio sobre el Manejo del Mielomeningocele.

**OBJETIVO:**

La cirugía materno-fetal abierta para el cierre intrauterino de mielomeningocele está asociada con beneficios desde la infancia hasta la edad escolar; sin embargo, los riesgos obstétricos y reproductivos maternos también son factores a considerar. El objetivo de este análisis fue evaluar los resultados reproductivos después de la cirugía materno-fetal abierta en comparación con el cierre postnatal estándar de mielomeningocele.

**DISEÑO DEL ESTUDIO:**

El Estudio sobre el Manejo del Mielomeningocele fue un ensayo aleatorizado que comparó el cierre prenatal y posnatal del mielomeningocele. Las mujeres del grupo de cierre prenatal se sometieron a cirugía materno-fetal abierta entre las semanas 21 y 26 de gestación y a cesárea a las 37 semanas si no habían tenido un parto antes. En el grupo de cierre posnatal, la cesárea se realizó a las 37 semanas y los neonatos se sometieron a cierre quirúrgico poco después del nacimiento. Las familias regresaron para seguimiento a los 30 meses y nuevamente a la edad escolar, entre los 6 y los 10 años. Se administraron cuestionarios reproductivos maternos durante las visitas de seguimiento para evaluar la menstruación, la fertilidad, los problemas ginecológicos y los resultados del embarazo posterior. Nosotros comparamos las variables continuas mediante la prueba de Wilcoxon y las variables categóricas mediante la prueba de chi-cuadrado o la prueba exacta de Fisher, según correspondiera.

**RESULTADOS:**

Un total de 174 mujeres aleatorizadas (86 en el grupo de cierre prenatal y 88 en el grupo de cierre posnatal) completaron cuestionarios reproductivos, donde 91 mujeres no reportaron embarazos posteriores desde el Estudio de Manejo del Mielomeningocele. En el grupo de cierre prenatal, 36 mujeres reportaron 60 embarazos posteriores, de los cuales 45 (75%) progresaron ≥20 semanas. En el grupo de cierre posnatal, 43 mujeres reportaron 71 embarazos, de los cuales 50 (70%) progresaron ≥20 semanas. El grupo de cierre prenatal tuvo mayor probabilidad de dar a luz antes de las 37 semanas (p < 0,001). Se reportaron una rotura uterina (2,2%) y dos dehiscencias uterinas (4,4%) en el grupo de cierre prenatal; ninguna en el grupo de cierre posnatal (p < 0,001). El uso de tratamientos de fertilidad o cirugías ginecológicas no fue diferente entre los grupos.

**CONCLUSIÓN:**

El parto prematuro fue más frecuente en embarazos posteriores a la cirugía materno-fetal abierta. El riesgo de rotura uterina y dehiscencia fue mayor en el grupo prenatal que en el posnatal, pero menor que lo reportado previamente con la cirugía materno-fetal abierta. Los resultados reproductivos fueron similares entre las mujeres que recibieron cirugía materno-fetal abierta para el cierre de mielomeningocele y las del grupo de cierre posnatal en el Estudio de Manejo del Mielomeningocele.

## Introduction

In 2011, the results of the Management of Myelomeningocele Study (MOMS) were published after the trial was stopped early on the basis of demonstrated efficacy.^1^ This multicenter randomized trial found that in utero closure of myelomeningocele (MMC) was associated with lower rates of ventriculoperitoneal shunting, higher likelihood of independent ambulation, reversal of hindbrain herniation, and improved motor function. The durability of the findings was confirmed by subsequent investigation of longer-term outcomes up to 10 years of age.^2-4^

Given the significant neonatal and childhood benefits, open maternal–fetal surgery (OMFS) for MMC closure has become a standard-of-care option for women carrying pregnancies prenatally diagnosed with spina bifida.^5^ Despite the benefits, clear OMFS for MMC closure carries clear risks, including obstetrical complications that lead to increased risk for preterm delivery, and complications of hysterotomy that can lead to uterine dehiscence or uterine rupture.^1^

The risk for uterine dehiscence and rupture remains a concern for subsequent pregnancies throughout the reproductive lifetime of the woman after undergoing OMFS. Uterine rupture can lead to catastrophic outcomes for both the mother and the infant. Previous reports suggest that the rates of uterine dehiscence and uterine rupture in subsequent pregnancies after OMFS range from 14% to 21% and 7% to 14%, respectively.^6-9^ The objective of this study is to evaluate maternal reproductive outcomes of patients in the MOMS trial and assess reproductive risk after OMFS compared with postnatal closure for MMC.

## Materials and methods

MOMS was a randomized trial comparing prenatal vs postnatal closure of MMC performed at 3 centers (Children’s Hospital of Philadelphia, Vanderbilt University Medical Center, and the University of California, San Francisco) and the Data Coordinating Center at the George Washington University Biostatistics Center.^1^ The trial enrollment was stopped early for efficacy after 183 maternal–fetal dyads were randomized. Women in the prenatal closure arm underwent OMFS at 21 to 26 weeks of gestation and underwent cesarean delivery at 37 weeks if they were not delivered before that time. In the postnatal closure arm, cesarean delivery was performed at 37 weeks, and neonates underwent surgical closure soon after birth. Families returned for follow-up at 30 months (MOMS) and again at school age, between 6 and 10 years (MOMS2).^1-3^ Institutional review board approval was obtained.

Maternal reproductive questionnaires were administered at the time of the follow-up visits (Supplemental Material). Participants were asked about whether they had tried to conceive or had any pregnancies since their MOMS pregnancy, as well as any complications or outcomes of the completed pregnancies. Restoration of menses, gynecologic procedures, use of infertility treatments, and desire for future pregnancies were also assessed. Patients were also asked for permission to access previous obstetrical records when available, and provided consent. Data from the reproductive questionnaires completed by each patient at the time most distant from the MOMS delivery were used for analysis. If the index MOMS child was known to have died, women were not approached to complete a reproductive questionnaire and were thus excluded.

### Statistical analysis

Patients were analyzed on the basis of their randomization status in MOMS to assess differences in reproductive outcomes between the groups and the correlation to the type of hysterotomy performed. Those randomized to prenatal closure received a hysterotomy in the active/fundal portion of the uterus, and those randomized to postnatal closure underwent routine cesarean delivery.^1^ We compared continuous variables using the Wilcoxon test and categorical variables using the chi-square or Fisher exact test, as appropriate. Nominal 2-sided P values of <.05 were considered to indicate statistical significance; no adjustments were made for multiple comparisons.

## Results

A total of 91 maternal–fetal dyads were randomly assigned to prenatal surgery (Figure), of which 1 woman refused prenatal surgery after randomization before any intervention, and 4 fetal deaths occurred in this group. A total of 92 maternal–fetal dyads were assigned to postnatal surgery (Figure). One family was lost to follow-up, and 3 neonates died before 30 months. Cases experiencing fetal or neonatal death or those lost to follow-up were excluded from this analysis. A total of 174 women were included in this analysis: 86 who underwent prenatal closure and 88 who underwent postnatal surgery and completed the reproductive questionnaire at 30 months. A total of 76 (88.4%) women in the prenatal group and 76 (86.4%) women in the postnatal group also completed the reproductive questionnaire at follow-up at 6 to 10 years (MOMS2). Among the patients providing reproductive questionnaire data in MOMS2, the visit occurred at a mean of 7.9 years (±1.2) after the MOMS delivery.

There were no differences in maternal demographics or reproductive status at enrollment in MOMS (Table 1). There were no differences in resumption of menses, desire to conceive a subsequent pregnancy, or actively trying to conceive a subsequent pregnancy between the prenatal and postnatal groups (Table 2).

Thirty-six women in the prenatal group had 60 subsequent pregnancies: 45 lasting ≥20 weeks (75.0%) and 15 lasting <20 weeks. Forty-three women in the postnatal group had 71 subsequent pregnancies: 50 lasting ≥20 weeks (70.4%) and 21 lasting <20 weeks. The number of pregnancies after MOMS and the number of losses <20 weeks were similar between the groups (Table 2). Preterm delivery in subsequent pregnancies occurred more often in the prenatal group than in the postnatal group. All of the infants delivered preterm were viable. Within the prenatal surgery group, 80% delivered at 36 weeks, 13% delivered at 35 weeks, 4% delivered at 34 weeks, and 2% delivered at 31 weeks.

There were no reported cases of uterine rupture or dehiscence in subsequent pregnancies in the postnatal group. In the prenatal group, there were 2 reported cases of uterine dehiscence (4.4%) and 1 case of uterine rupture (2.2%) (*P*<.001). The 2 uterine dehiscence cases occurred in the first subsequent pregnancy after MOMS, delivering at 36 weeks and 5 days and 36 weeks and 3 days. Both were asymptomatic, diagnosed at the time of delivery at the prior OMFS site, with a pregnancy interval of 2 years. The uterine rupture occurred at 31 weeks of gestation in a first subsequent pregnancy 5 years after the MOMS delivery at the OMFS site. Maternal and neonatal outcomes were favorable in all 3 cases. No placental concerns were reported in these cases.

The use of infertility therapy at the time of enrollment in the MOMS trial was 11.6% in the prenatal surgery group and 14.8% in the postnatal group, and this difference was not statistically significant (*P*=.656). These rates were also not statistically significantly different between the groups at the 30-month and 6- to 10-year follow-up (Table 3).

Any gynecologic or abdominal surgical procedures, including subsequent cesarean delivery, occurred in 60.5% of prenatal surgery patients and 56.6% of postnatal patients, and this difference was not statistically significant (*P*=.74) (Table 3). The rates of hysterectomy, appendectomy, dilation and curettage, treatment for abnormal Papanicolaou smear, laparoscopy, or intestinal surgery were not statistically significantly different between the prenatal and postnatal groups. One patient in the prenatal surgery group, who had an asymptomatic uterine dehiscence during the MOMS pregnancy, underwent hysterotomy revision as an elective interval gynecologic procedure, not associated with a pregnancy. The rate of tubal ligation was not different between the groups.

## Comment

### Principal findings

According to the results of reproductive questionnaires from women enrolled in the MOMS trial, the rates were 2.2% for uterine rupture after OMFS for MMC closure and 4.4% for uterine dehiscence. Women who underwent OMFS were at increased risk for preterm delivery in subsequent pregnancies. OMFS did not appear to impact fertility or gynecologic outcomes.

### Results in the context of what is known

Maternal reproductive questionnaire responses from the women enrolled in the MOMS trial provide a comprehensive assessment of long-term subsequent pregnancy and reproductive outcomes. The impact of a hysterotomy in the active portion of the uterus associated with OMFS raises concern for poor subsequent pregnancy outcomes, including uterine rupture and dehiscence. The rates of uterine rupture (2.2%) and uterine dehiscence (4.4%) in the MOMS cohort of women undergoing OMFS were lower than the previously reported rates of 7% to 14% for uterine rupture and 14% to 21% for uterine dehiscence.^6-9^ This may be due to reporting bias or low numbers of subsequent pregnancies in other studies. In the study by Wilson et al,^6^ women who underwent OMFS at a single institution for multiple indications, including MMC closure, sacrococcygeal teratoma debulking, and resection of fetal lung lesions, among others, were sent questionnaires regarding reproductive outcomes. Their response rate was 57%. Many of these OMFS procedures involved larger hysterotomies than are typically used for MMC closure given the indication for the procedure. The study by Goodnight et al^7^ reports on a large number of subsequent pregnancies after OMFS for MMC closure from a number of centers. The data were derived from a multicenter registry, which inherently introduces potential bias in data acquisition. Two more recent studies report uterine rupture rates of 7% and 10% from single centers.^8,9^ Data from both studies were obtained using reproductive questionnaires, with return rates of approximately 70% in each, as opposed to over 85% completion up to 6 to 10 years after OMFS in this cohort.

The current combined uterine rupture/dehiscence rate of 6.6% is likely a much more realistic rate than has been cited elsewhere given the deliberate nature of acquisition of subsequent pregnancy outcomes embedded into the long-term follow-up of the MOMS trial. Notably, the uterine rupture rate associated with classical cesarean delivery is reported to be in the range of 4% to 9%.^10,11^ Another large cross-sectional study comparing prior classical cesarean delivery and low transverse cesarean delivery reported a rate of uterine rupture of 10.6% among women who underwent labor as opposed to 0.3% among women who did not.^12^ Asymptomatic uterine dehiscence was noted in 9% of prior classical cesarean deliveries in one study.^11^ Therefore, in the current OMFS cohort, the risk of uterine rupture/dehiscence in subsequent pregnancies after OMFS was similar to that following classical cesarean delivery. This is an important consideration in counseling women who are candidates for OMFS for MMC closure. It is possible that, with improvement in minimally invasive fetoscopic techniques for MMC closure, subsequent pregnancy outcomes might involve decreased risk, as suggested by a theoretical model comparing OMFS with fetoscopic methods.^13^

Furthermore, when the MOMS trial was developed, cesarean delivery was chosen as the mode of delivery even in cases of postnatal closure to reduce confounding factors. Recent studies have shown that there may be no benefit to cesarean delivery over vaginal delivery with regard to neurologic function in cases of fetal spina bifida.^14,15^ This paradigm change in obstetrical management of pregnancies complicated by fetal spina bifida may further alter maternal reproductive risk by supporting vaginal delivery.

The recommended timing of delivery after OMFS is 36 to 37 weeks.^7^ This is similar to recommendations for the timing of delivery after prior classical cesarean delivery, myomectomy, or uterine rupture.^10-12,16^ There were no definitive factors associated with uterine rupture in a large series of subsequent pregnancies after OMFS, including location of hysterotomy, interdelivery interval, or appearance of hysterotomy at delivery of the OMFS pregnancy.^7^ However, the general recommendation remains to achieve an interdelivery interval of >24 months to minimize the risk of uterine rupture, on the basis of data from studies of trial of labor after cesarean delivery.^17,18^

In this cohort, the prenatal surgery group had a higher proportion of preterm deliveries in subsequent pregnancies than the postnatal group. This is largely presumed to be due to the recommendation for delivery at 36 to 37 weeks after OMFS.^7,10-12,16^ The rate of preterm birth in prior studies is 22% to 31%.^6,8,9^ The average gestational age at delivery has been reported to be 36 to 37 weeks.^6-8^ In the current cohort, 80% of the preterm deliveries occurred at 36 weeks, consistent with clinical recommendations.

There was no difference between the groups with regard to early pregnancy loss. The current rate of spontaneous miscarriage of 12.8% is similar to the 11.4% rate reported by Zepf et al,^8^ but lower than the 25% reported in another 9 study.^9^

In this study, resumption of regular menses did not differ between the prenatal and postnatal groups. In one study, 40% of patients reported abnormal menstrual bleeding after OMFS.^9^ This is in contrast to the nearly 70% of patients in the current cohort who reported resumption of normal menses.

The desire for future pregnancies did not differ between the prenatal and postnatal groups. In both groups, 2% to 7% desired an additional pregnancy or were actively attempting to conceive. Similarly, one study reported that over 82% of patients did not attempt further conception, linking the decision to avoid pregnancy to the events of the index pregnancy.^9^ The current study did not investigate this aspect of decision-making.

The conclusion of a prior report was that infertility and untoward gynecologic outcomes were not increased among women undergoing OMFS.^6^ There was no difference in the use of infertility treatments or gynecologic/abdominal surgical interventions between the prenatal and postnatal groups in the post-MOMS cohort. The rate of in vitro fertilization (IVF) use was 9.2% in the prenatal surgery group at 6 to 10 years after MOMS. The rate of IVF use was 11.6% for the index MOMS pregnancy, suggesting that the OMFS during the study did not impact fertility in this group. The rates of IVF use after OMFS for fetal MMC closure in other studies are similarly reported at 6.9% to 8%.^8,9^ This provides further support that OMFS does not appear to impact gynecologic function or fertility.

### Clinical implications

Women carrying a fetus diagnosed with spina bifida who meet criteria for in utero closure must weigh the risks and benefits of OMFS for the current pregnancy, as well as subsequent pregnancies and their own reproductive health. According to these data, the risk for uterine rupture/dehiscence in a subsequent pregnancy after OMFS is similar to that following classical cesarean delivery, which is commonly managed by obstetrical providers globally. This finding should be reassuring to women seeking to optimize long-term outcomes for their fetus. Additionally, OMFS for MMC closure does not seem to impact maternal reproductive health.

### Research implications

This research does not capture the entirety of some of the mothers’ reproductive years, underscoring the value of continued follow-up of this cohort. Comparing these maternal outcomes with outcomes after fetoscopic repair may help pregnant women in their treatment decisions.

### Strengths and limitations

This study has many strengths, including the prospective data collection within a randomized trial. Only 8 women who entered randomization did not provide any reproductive questionnaire feedback, with over 86% of women in each group providing responses up to 6 to 10 years after OMFS in the MOMS trial. This allowed for a robust analysis of reproductive outcomes in this cohort, followed for up to 10 years, and indicates a high capture rate of subsequent pregnancies. The ~14% of patients in both the prenatal and postnatal groups who did not provide reproductive questionnaire responses at the 6- to 10-year follow-up, although equivalent, represent a limitation because this leaves the possibility that untoward outcomes were underreported. Furthermore, maternal reproductive outcomes will be assessed in the ongoing MOMS3 study, which will evaluate participants as adolescents and young adults, from ages of 15 to 24 years.

### Conclusion

Analysis of subsequent pregnancies after OMFS for fetal MMC closure in the MOMS trial revealed a combined uterine rupture and dehiscence rate of 6.6%. This rate is lower than previously reported with OMFS but similar to rates associated with prior classical cesarean delivery, and patients should be counseled accordingly. Subsequent pregnancies are at increased risk for preterm delivery after OMFS.

## Figures and Tables

**FIGURE:**
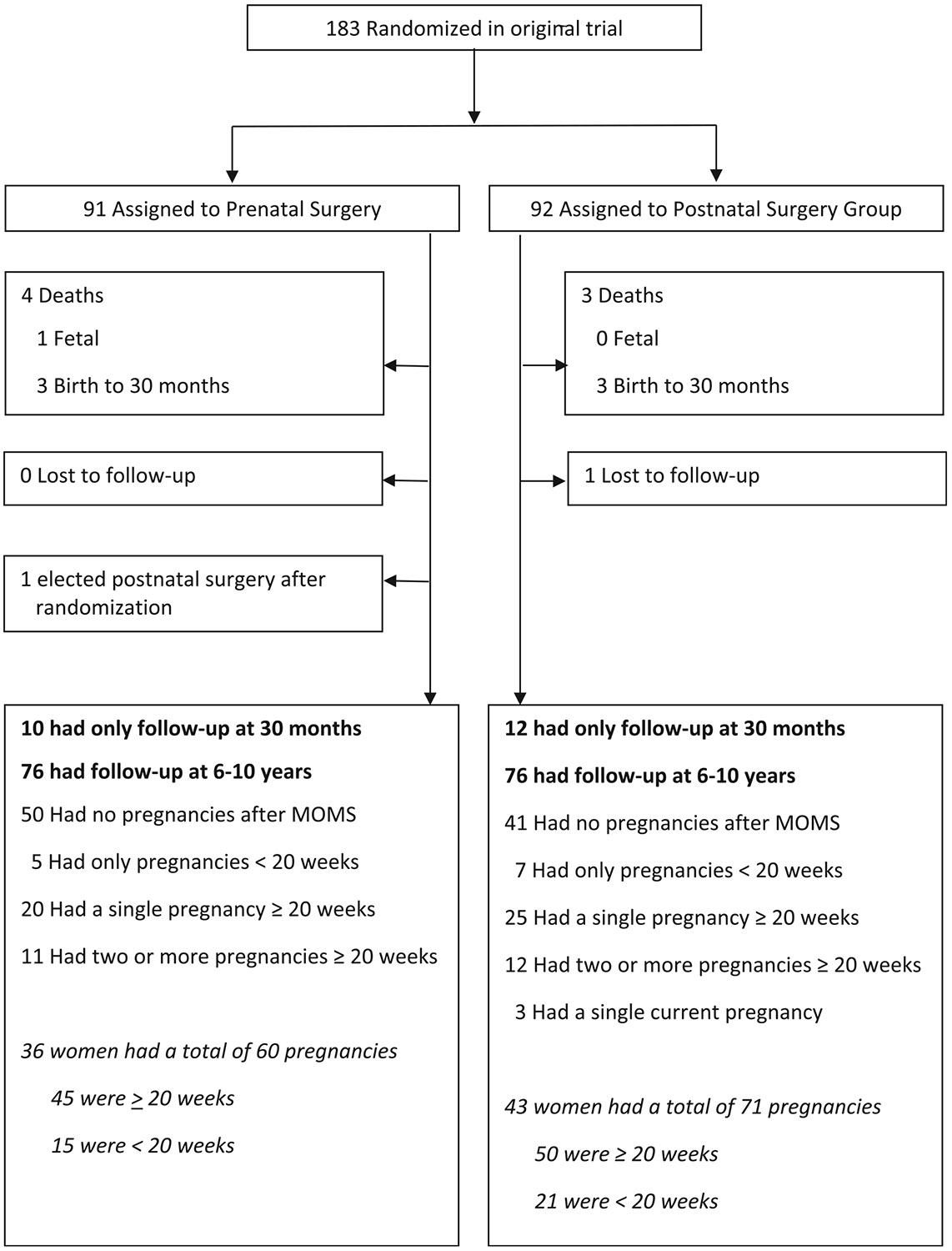
Enrollment and follow-up

**TABLE 1:** Maternal demographics and reproductive status at enrollment into MOMS

Characteristics	Prenatal surgery N=86	Postnatal surgery N=88	*P* value
Maternal age (y)	29.3 (29–41)	28.7 (18–41)	.53
BMI	26.3 (19.7–33.8)	26.3 (18.2–34.5)	.80
Total years of school	15.0 (11–17)	15.0 (12–17)	.80
Race			.87
White	84 (97.7%)	83 (94.3%)	
African American	1 (1.2%)	1 (1.1%)	
Asian	0	1 (1.1%)	
American Indian/Alaskan Native	1 (1.2%)	1 (1.1%)	
Other	0	2 (2.3%)	
Hispanic	2 (2.3%)	4 (4.6%)	.68
Married	80 (93.0%)	82 (93.2%)	.97
Smoker	4 (4.7%)	5 (5.7%)	1.00
MOMS pregnancy=first pregnancy	30 (34.9%)	29 (33.0%)	.79
Nulliparous at MOMS enrollment	35 (40.7%)	35 (39.8%)	.90
Number of prior live births			.53
0	36 (41.9%)	35 (39.8%)	
1	20 (23.3%)	29 (33.0%)	
2	21 (24.4%)	18 (20.5%)	
3	6 (7.0%)	4 (4.6%)	
4	3 (3.5%)	1 (1.1%)	
7	0	1 (1.1%)	
History of preeclampsia	2 (2.3%)	3 (3.4%)	1.00
History of cesarean delivery			.69
0	74 (86.1%)	77 (87.5%)	
1	8 (9.3%)	9 (10.2%)	
2	4 (4.7%)	2 (2.3%)	
History of any uterine surgery	12 (14.0%)	11 (12.5%)	.78
History of miscarriage <20 wk			.23
0	61 (70.9%)	68 (77.3%)	
1	19 (22.1%)	11 (12.5%)	
2	6 (7.0%)	9 (10.2%)	
Location of hysterotomy at fetal surgery			—
Anterior	40 (46.5%)	n/a	
Anterior fundal	3 (3.5%)	n/a	
Fundal	7 (8.1%)	n/a	
Posterior	25 (29.1%)	n/a	
Posterior fundal	11 (12.8%)	n/a	

*BMI*, body mass index; *MOMS*, Management of Myelomeningocele Study.

**TABLE 2:** Reproductive status after MOMS

Characteristics of reproductive status	Prenatal N=86	Postnatal N=88	*P* value
Length of follow-up			
30-mo visit only (N)	10	12	
Visit at 6–10 y of age (N)	76	76	
Child age at 6- to 10-y visit, mean (SD)	7.9 (1.2)	7.9 (1.3)	
Resumption of menses			.50
Not resumed	5 (5.8%)	2 (2.3%)	
Resumed irregular	22 (25.6%)	22 (25.0%)	
Resumed regular	59 (68.6%)	64 (72.7%)	
Attempting to conceive			.31
Currently pregnant	2 (2.3%)	8 (9.1%)	
Sterilized	28 (32.6%)	25 (28.4%)	
Not trying to conceive	54 (62.8%)	53 (60.2%)	
Trying to conceive	2 (2.3%)	2 (2.3%)	
Desire pregnancy, if not trying to conceive?	N=54	N=52	.81
No	37 (68.5%)	34 (65.4%)	
Not certain	13 (24.1%)	15 (28.9%)	
Yes	4 (7.4%)	3 (5.8%)	
Total number of pregnancies since MOMS			.79
0	50 (58.1%)	44 (50.0%)	
1	21 (24.4%)	25 (28.4%)	
2	10 (11.6%)	13 (14.8%)	
3	2 (2.3%)	4 (4.6%)	
4	2 (2.3%)	2 (2.3%)	
5	1 (1.2%)	0	
	60 pregnancies total	71 pregnancies total	
Losses <20 wk			.45
0	75 (87.2%)	71 (80.7%)	
1	7 (8.1%)	13 (14.8%)	
2	4 (4.7%)	4 (4.6%)	
Preterm delivery 20–36 wk			<.001
0	69 (80.2%)	86 (97.7%)	
1	15 (17.4%)	1 (1.1%)	
2	2 (2.3%)	1 (1.1%)	
Delivery ≥37 wk			.04
0	67 (77.9%)	53 (60.2%)	
1	12 (14.0%)	24 (27.3%)	
2	7 (8.1%)	10 (11.4%)	
3	0	1 (1.1%)	

*MOMS*, Management of Myelomeningocele Study.

**TABLE 3:** Use of infertility treatments, gynecologic, and abdominal surgery after MOMS

Treatment types	Prenatal	Postnatal	*P* value
Infertility treatment before MOMS pregnancy	10/86 (11.6%)	13/88 (14.8%)	.66
Infertility treatment at 30 mo	2/86 (2.33%)	1/88 (1.14%)	.62
Infertility treatment at 6–10 y	7/76 (9.21%)	2/75 (2.67%)	.17
Hysterectomy	9/86 (10.5%)	5/88 (5.7%)	.28
Appendectomy	2/86 (2.3%)	2/88 (2.3%)	1.00
Laparoscopy	8/86 (9.3%)	5/88 (5.7%)	.40
Intestinal surgery	1/86 (1.2%)	0	.49
D&C	9/86 (10.5%)	3/88 (3.4%)	.08
Treatment for abnormal Papanicolaou smear	7/86 (8.1%)	4/88 (4.6%)	.37
Sterilization procedure/tubal ligation	8/86 (9.3%)	10/88 (11.4%)	.80

*D&C*, dilation and curettage; *MOMS*, Management of Myelomeningocele Study.

## AJOG MFM at a Glance

### Why was this study conducted?

Maternal reproductive questionnaires were obtained at childhood follow-up over time for women involved in the Management of Myelomeningocele Study trial to investigate long-term reproductive outcomes and subsequent pregnancy outcomes after open maternal–fetal surgery (OMFS).

### Key findings

Preterm delivery was more common in subsequent pregnancies after OMFS. The uterine rupture and dehiscence rate in subsequent pregnancies after OMFS was lower than previously reported.

### What does this add to what is known?

Women should be counseled that the risk for uterine rupture/dehiscence in subsequent pregnancies after OMFS for spina bifida closure is lower than previously reported but remains a concern.

## References

[1] Adzick NS, Thom EA, Spong CY, A randomized trial of prenatal versus postnatal repair of myelomeningocele. N Engl J Med 2011;364:993–1004. 21306277 10.1056/NEJMoa1014379 PMC3770179

[2] Farmer DL, Thom EA, Brock III JW, The Management of Myelomeningocele Study: full cohort 30-month pediatric outcomes. Am J Obstet Gynecol 2018;218:256.e1–256.e13. 10.1016/j.ajog.2017.12.001 PMC7737375 29246577

[3] Houtrow AJ, Thom EA, Fletcher JM, Prenatal repair of myelomeningocele and school-age functional outcomes. Pediatrics 2020;145:e20191544. 31980545 10.1542/peds.2019-1544 PMC6993457

[4] Houtrow AJ, MacPherson C, Jackson-Coty J, Prenatal repair and physical functioning among children with myelomeningocele: a secondary analysis of a randomized clinical trial. JAMA Pediatr 2021;175:e205674. 33555337 10.1001/jamapediatrics.2020.5674 PMC7871205

[5] Committee Opinion No. 720: Maternal-fetal surgery for myelomeningocele. Obstet Gynecol 2017;130:e164–7. 28832491 10.1097/AOG.0000000000002303

[6] Wilson RD, Lemerand K, Johnson MP, Reproductive outcomes in subsequent pregnancies after a pregnancy complicated by open maternal-fetal surgery (1996–2007). Am J Obstet Gynecol 2010;203:209.e1–6. 10.1016/j.ajog.2010.03.029 20537307

[7] Goodnight WH, Bahtiyar O, Bennett KA, Subsequent pregnancy outcomes after open maternal-fetal surgery for myelomeningocele. Am J Obstet Gynecol 2019;220:494.e1–7. 10.1016/j.ajog.2019.03.008 PMC6511319 30885769

[8] Zepf J, Vonzun L, Kräahenmann F, Subsequent pregnancy outcomes after open in utero spina bifida repair. Fetal Diagn Ther 2022;49:442–50. 36455544 10.1159/000527813

[9] Haenen K, Vergote S, Kunpalin Y, Subsequent fertility, pregnancy, and gynaecological and psychological outcomes after maternal-fetal surgery for open spina bifida: a prospective cohort study. BJOG 2023;130:1677–84. 10.1111/1471-0528.17557. 37272251

[10] Landon MB, Lynch CD. Optimal timing and mode of delivery after cesarean with previous classical incision or myomectomy: a review of the data. Semin Perinatol 2011;35:257–61. 21962624 10.1053/j.semperi.2011.05.008

[11] Chauhan SP, Magann EF, Wiggs CD, Barrilleaux PS, Martin JN Jr. Pregnancy after classic cesarean delivery. Obstet Gynecol 2002;100:946–50. 12423858 10.1016/s0029-7844(02)02239-1

[12] Thompson BB, Reddy UM, Burn M, Abdel-Razeq S, Xu X. Maternal outcomes in subsequent pregnancies after classical cesarean delivery. Obstet Gynecol 2022;140:212–9. 35852271 10.1097/AOG.0000000000004869

[13] Packer CH, Hersh AR, Caughey AB. Fetoscopic compared with open repair of myelomeningocele: a 2-delivery cost-effectiveness analysis. Am J Obstet Gynecol MFM 2021;3:100434. 34217856 10.1016/j.ajogmf.2021.100434

[14] Tolcher MC, Shazly SA, Shamshirsaz AA, Neurological outcomes by mode of delivery for fetuses with open neural tube defects: a systematic review and meta-analysis. BJOG 2019;126:322–7. 29924919 10.1111/1471-0528.15342

[15] Palatnik A, Pan AY, Emery SP, Mode of delivery and subsequent motor function in children with myelomeningocele without in utero repair. Obstet Gynecol 2025;145:316–23. 39820255 10.1097/AOG.0000000000005823

[16] American College of Obstetricians and Gynecologists’ Committee on Obstetric Practice. Society for Maternal-Fetal Medicine. Medically indicated late-preterm and early-term deliveries: ACOG committee opinion, Number 831. Obstet Gynecol 2021;138:e35–9. 34259491 10.1097/AOG.0000000000004447

[17] Shipp TD, Zelop CM, Repke JT, cohen A, Lieberman E. Interdelivery interval and risk of symptomatic uterine rupture. Obstet Gynecol 2001;97:175–7. 11165577 10.1016/s0029-7844(00)01129-7

[18] Bujold E, Gauthier RJ. Risk of uterine rupture associated with an interdelivery interval between 18 and 24 months. Obstet Gynecol 2010;115:1003–6. 20410775 10.1097/AOG.0b013e3181d992fb

